# 405例肺癌合并肺结核患者临床特征及驱动基因检测分析

**DOI:** 10.3779/j.issn.1009-3419.2020.101.25

**Published:** 2020-05-20

**Authors:** 瑛 胡, 新杰 杨, 理会 聂, 丹 赵, 军 安, 宝兰 李

**Affiliations:** 1 101149 北京, 首都医科大学附属北京胸科医院肿瘤内科 Department of Oncology, Beijing Chest Hospital, Capital Medical University, Beijing 101149, China; 2 101149 北京, 首都医科大学附属北京胸科医院结核内科 Department of Tuberculosis, Beijing Chest Hospital, Capital Medical University, Beijing 101149, China; 3 101149 北京, 首都医科大学附属北京胸科医院病理科 Department of Pathology, Beijing Chest Hospital, Capital Medical University, Beijing 101149, China; 4 101149 北京, 首都医科大学附属北京胸科医院病案科 Department of Medical Records, Beijing Chest Hospital, Capital Medical University, Beijing 101149, China

**Keywords:** 肺肿瘤, 肺结核, 驱动基因, Lung neoplasms, Pulmonary tuberculosis, Driver gene

## Abstract

**背景与目的:**

随着肺癌研究进展, 靶向治疗、免疫检查点抑制剂等新的治疗方法已经开始应用于肺癌患者, 因此需要进一步了解合并肺结核的肺癌患者的临床及实验室特点, 从而为此类患者的临床治疗提供新的思路。本研究目的是分析肺癌合并肺结核患者的临床特征、驱动基因检测结果及其之间关系。

**方法:**

回顾性分析我院2014年1月-2019年12月收治的405例肺癌合并肺结核患者, 应用统计学方法分析其临床特征与驱动基因状态之间的关系。

**结果:**

405例肺癌合并肺结核患者中男性占77.3%, 有吸烟史患者占85.3%, 病理类型以肺腺癌为主, 当胸部影像学有空洞改变时以鳞癌为主要类型。214例患者进行驱动基因检测, 表皮生长因子受体(epidermal growth factor receptor, *EGFR*)基因突变率为35.9%, 其中41.8%为外显子19缺失突变, 50.9%为外显子21 L858R突变。当胸部影像有空洞改变时, *EGFR*突变率显著降低(16.1%)。间变性淋巴瘤激酶(anaplastic lymphoma kinase, *ALK*)融合基因检测阳性率为2.5%, 原癌基因1酪氨酸激酶(c-ros oncogene 1 receptor kinase, *ROS1*)突变率为1.9%, 肉瘤病毒致癌基因同源物B1(V-raf murine sarcoma viral oncogene homolog B1, BRAF)基因突变率为1.1%, 克尔斯滕大鼠肉瘤病毒致癌基因同源物(Kirsten Rat Sarcoma Viral Oncogene Homolog, *KRAS*)基因突变率为10.1%。女性肺癌合并肺结核患者基因突变率为50.0%, 男性为27.9%。

**结论:**

肺癌合并肺结核患者以有吸烟史的男性患者为主, 病理类型以腺癌为主。基因突变阳性率与单纯肺癌无明显差异, 但是当胸部影像有空洞表现时, 基因突变率显著降低。

## Abstract (en)

**Background and objective:**

New treatment methods such as targeted therapy and immune checkpoint inhibitors have been applied to lung cancer patients. It is necessary to further understand the patients with lung cancer combined with pulmonary tuberculosis with the development of lung cancer research. The purpose of this study was to analyze the clinical characteristics of lung cancer patients with pulmonary tuberculosis, the status of driver genes, and their relationships.

**Methods:**

A retrospective analysis was performed on 405 patients with lung cancer and pulmonary tuberculosis hospitalized in our hospital from January 2014 to December 2019. The relationship between clinical characteristics and driver genes status was analyzed.

**Results:**

Among the 405 patients with lung cancer combined with pulmonary tuberculosis, 77.3% were male and 85.3% were patients with a history of smoking. The pathological type was mainly lung adenocarcinoma. When there were cavities in chest computed tomography (CT), squamous cell carcinoma was the main type. 214 patients underwent driver genes testing. The epidermal growth factor receptor (*EGFR*) gene mutation rate was 35.9%, of which 41.8% were exon 19 deletion mutations and 50.9% were exon 21 L858R mutations. When there were cavities in the chest CT, the *EGFR* mutation rate was significantly reduced (16.1%). The positive rate of anaplastic lymphoma kinase (*ALK*) fusion gene detection was 2.5%, the mutation rate of c-ros oncogene 1 receptor kinase (*ROS1*) gene was 1.9%, the mutation rate of V-raf murine sarcoma viral oncogene homolog B1 (*BRAF*) gene was 1.1%, and the mutation rate of Kirsten Rat Sarcoma Viral Oncogene Homolog (*KRAS*) gene was 10.1%. The genetic mutation rate of female patients with lung cancer and pulmonary tuberculosis was 50.0%, and that of men was 27.9%.

**Conclusion:**

Patients with lung cancer and pulmonary tuberculosis are predominantly male with smoking history. Adenocarcinoma is the most common pathological type. The positive rate of gene mutation was not significantly different from that of simple lung cancer, but when there were cavities in the chest image, the genetic mutation rate was significantly reduced.

肺结核患者的肺癌发病率是无肺结核人群肺癌发病率的10.9倍, 合并肺结核的肺癌患者死亡率是单纯肺癌患者死亡率的8倍^[1]^。目前肺癌发病率及死亡率均为全球癌症发病率及死亡率之首, 肺癌的治疗方法在近十年发生巨大变化, 肺癌靶向治疗、免疫检查点抑制剂等新的治疗方法已经开始应用于肺癌患者。肿瘤科医生做肺癌相关治疗时, 需关注肺癌合并肺结核患者治疗方案的选择问题。需要进一步了解合并肺结核的肺癌患者的临床及实验室特点, 从而为此类患者的临床治疗提供新的思路。因此, 本研究旨在通过回顾合并肺结核的肺癌患者的临床资料, 分析患者的临床特征、影像学特点、基因突变情况及其之间关系, 从而为制定合并肺结核的肺癌患者的临床治疗方案及进一步开展相关研究, 提供有价值的参考依据。

## 1 资料和方法

### 1.1 研究对象

回顾性分析自2014年1月-2019年12月于首都医科大学附属北京胸科医院出院, 诊断中同时包含肺癌及肺结核的病例744例。参照纳入及排除标准, 符合本研究的共405例。

### 1.2 纳入标准

① 患者经肺穿刺活检、手术病理组织、淋巴结活检、胸水包埋等明确肺癌诊断。②患者在肺癌诊断之前, 有明确结核病诊断, 或于诊断肺癌同时, 诊断肺结核病。肺结核病诊断符合2018版肺结核诊断标准^[2]^。

### 1.3 排除标准

肺结核诊断时间晚于肺癌诊断时间。

### 1.4 肺癌分期分组

Ⅰ期-Ⅲa期为早期, Ⅲb期-Ⅳ期为局部晚期及进展期。

### 1.5 患者驱动基因检测

驱动基因检测均为首都医科大学附属北京胸科医院病理科操作完成, 应用突变扩增系统(amplification refractory mutation system, ARMS)荧光定量聚合酶链反应(polymerase chain reaction, PCR)方法检测。间变性淋巴瘤激酶(anaplastic lymphoma kinase, *ALK*)融合突变判定阳性的标准, 需要VENTANA全自动免疫组化法(immunohistochemistry, IHC)及ARMS荧光定量PCR均为阳性。

### 1.6 统计学处理

采用SPSS 22.0统计软件进行统计分析, 计数资料率的比较采用卡方检验, 年龄以均数±标准差表示, 行方差分析, 以*P* < 0.05表示差异具有统计学意义。

## 2 结果

### 2.1 肺癌合并肺结核患者资料

本研究回顾性分析405例肺癌合并肺结核患者临床资料, 平均年龄为(63.4±0.525)岁, 男性患者313例(77.3%), 女性患者92例(22.7%), 男女比例为3.4:1, 有吸烟史患者281例(69.4%), 男性患者中有吸烟史者为85.3%(267/313), 女性患者中为15.2%(14/92), 不同性别组间比较吸烟史差异显著(χ^2^=164.408, *P* < 0.000, 1)。405例患者确诊肺癌诊断时, 23.2%(94例)为活动性肺结核患者, 76.8%(311例)为陈旧性肺结核患者。

本研究中, 进行基因检测的患者为214例(52.8%)。分析214例患者与405例患者的性别、年龄、吸烟史及结核活动状态等基本临床资料, 均无统计学差异(*P* > 0.05)(表 1)。

**1:** 肺癌合并肺结核患者的基本临床资料 Basic clinical data of lung cancer patients with tuberculosis

Variables	All patients (*n*=405)	Patients driver genes status (*n*=214)	*χ*^2^	*P*
Gender			2.14	0.169
Male	313 (77.3%)	154 (72.0%)		
Female	92 (22.7%)	60 (28.0%)		
Age (yr)	63.4±0.525	63.96±0.678		0.956
Smoking history			0.788	0.414
Yes	281 (69.4%)	141 (65.9%)		
No	124 (30.6%)	73 (34.1%)		
Status of tuberculosis			0.092	0.766
Active	94 (23.2%)	52 (24.3%)		
Obsolete	311 (76.8%)	162 (75.7%)		

### 2.2 Ⅲb期-Ⅳ期肺癌患者合并活动性肺结核比例高于Ⅰ期-Ⅲa期患者

405例肺癌合并肺结核患者中, Ⅰ期-Ⅲa期为45.7%(185例), Ⅲb期-Ⅳ期为39.3%(159例), 未完成分期的患者为15.1%(61例)。Ⅰ期-Ⅲa期患者中, 活动性肺结核患者为14.1%(26例), 陈旧性肺结核患者为85.9%(159例), Ⅲb期-Ⅳ期患者中, 活动性肺结核患者为26.4%(42例), 陈旧性肺结核患者为73.6%(117例), 两组比较有显著性差异(χ^2^=8.238, *P*=0.004)。

405例肺癌合并肺结核患者的病理类型：肺腺癌占50.1%(203例), 肺鳞癌占32.3%(131例), 小细胞肺癌占9.9%(40例), 腺鳞癌占2.7%(11例), 肺大细胞癌占0.5%(2例), 类癌等其他神经内分泌肿瘤占2.2%(9例), 未分类的非小细胞肺癌占2.2%(9例)。

本研究中24.2%(98/405)的患者胸部影像学有空洞改变, 其中45.9%(45/98)为鳞癌, 36.7%(36/98)为腺癌; 75.8%(307/405)的患者胸部影像无空洞改变, 其中54.4%为腺癌(167/307), 28%为鳞癌(86/307), 两组间比较差异显著(χ^2^=11.968, *P*=0.001)。

### 2.3 肺癌合并肺结核患者基因突变状态

405例患者中, 214例(52.8%)进行了基因检测, 均为非小细胞肺癌患者, 其中鳞癌48例(22.4%), 非鳞癌166例(77.6%)。基因检测结果为：表皮生长因子受体(epidermal growth factor receptor, *EGFR*)基因突变率为35.9%(55/153), 其中外显子19缺失突变为41.8%(23/55), 外显子21 L858R突变为50.9%(28/55), 少见突变4例(7.3%), 分别为S768I、G719X/L861Q、L861Q及G719X/S768I。*ALK*融合基因检测阳性率为2.5%(5/199), 克尔斯滕大鼠肉瘤病毒致癌基因同源物(Kirsten Rat Sarcoma Viral Oncogene Homolog, *KRAS*)基因突变率为10.1%(12/119), 原癌基因1酪氨酸激酶(c-ros oncogene 1 receptor kinase, *ROS1*)基因突变率为1.9%(2/108), 肉瘤病毒致癌基因同源物B1(V-raf murine sarcoma viral oncogene homolog B1, *BRAF*)基因突变率为1.1%(1/88)(表 2)。

**2:** 214例肺癌合并肺结核患者基因检测情况 Genetic testing of 214 patients with lung cancer complicated with tuberculosis

Variables	Number of cases	Number of positive cases
*EGFR* mutation testing	153	55 (35.9%)
19DEL		23 (41.8%)
L858R		28 (50.9%)
Rare mutation		4 (7.3%)
*ALK* fusion mutation	199	5 (2.5%)
*KRAS* mutation testihng	119	12 (10.1%)
*ROS-1* mutation testihng	108	2 (1.9%)
*BRAF* mutation testihng	88	1 (1.1%)
EGFR: epidermal growth factor receptor; ALK: anaplastic lymphoma kinase; KRAS: kirsten Rat Sarcoma Viral Oncogene Homolog; ROS1: c-ros oncogene 1 receptor kinase; BRAF: V-raf murine sarcoma viral oncogene homolog B1.		

### 2.4 基因突变状态与性别、吸烟史、病理类型及胸部影像的空洞改变有关

214例患者临床特征与基因检测结果分析, 女性患者基因突变阳性率为50%(30/60), 而男性患者为27.9%(43/154), 两组比较有显著统计学差异(χ^2^=9.364, *P*=0.002)。有吸烟史患者基因突变阳性率为28.4%(40/141), 无吸烟史患者为45.2%(33/73), 组间比较差异显著(χ^2^=6.066, *P*=0.014)。腺癌患者基因突变率为45.2%(70/155), 56例鳞癌患者中, 仅4例患者存在基因突变, 占鳞癌患者的7.1%, 统计学分析显示, 腺癌与非腺癌患者组间比较, 鳞癌与非鳞癌患者组间比较, 基因突变阳性率均有显著统计学差异(*P* < 0.000, 1)(表 3)。

**3:** 214例肺癌合并肺结核患者基因突变情况与临床特征分析 Analysis of gene mutations and clinical characteristics in 214 patients with lung cancer complicated with tuberculosis

	Positive mutation (*n*=73)	Negative mutation (*n*=141)	*χ*^2^	*P*
Gender			9.364	0.002
Male	43 (27.9%)	111 (72.1%)		
Female	30 (50.0%)	30 (50.0%)		
Age (yr)	63.49±1.314	64.2±0.776		0.922
Smoking history			6.066	0.014
Yes	40 (28.4%)	101 (71.6%)		
No	33 (45.2%)	40 (54.8%)		
Status of tuberculosis			0.848	0.357
Active	15 (28.8%)	37 (71.2%)		
Obsolete	58 (25.8%)	104 (64.2%)		
Tumor stage			2.947	0.086^#^
Ⅰ-Ⅲa	25 (29.1%)	61 (70.9%)		
Ⅲb-Ⅳ	45 (40.9%)	65 (59.1%)		
Indefinite	3 (16.7%)	15 (83.3%)		
Pathological types				
Adenocarcinoma (with mixed adenocarcinoma)	70 (45.2%)	85 (54.8%)	30.538	< 0.0001
Non-adenocarcinoma	3 (5.1%)	56 (94.9%)		
Squamous carcinoma (with mixed squamous carcinoma)	4 (7.1%)	52 (92.9%)	24.545	< 0.0001
Non-squamous carcinoma	69 (43.7%)	89 (56.3%)		
Cavities in chest imaging				
Yes	10 (21.3%)	37 (78.7%)	4.415	0.036
Squamous carcinoma	1 (5.3%)	18 (94.7%)		0.034^*^
Non-squamous carcinoma	9 (32.1%)	19 (67.9%)		
No	63 (37.7%)	104 (62.3%)		
Squamous carcinoma	3 (8.1%)	34 (91.9%)	17.746	< 0.0001
Non-squamous carcinoma	60 (46.2%)	70 (53.8%)		
^#^Statistical analysis was performed on the two groups of stage Ⅰ-Ⅲa and Ⅲb-Ⅳ. ^*^Fisher’s exact test.				

胸部CT有无空洞改变与基因突变状态之间关系分析, 本研究中伴有空洞改变并完成基因突变检测的患者共47例, 其中31例行*EGFR*基因检测, 5例(16.1%)为阳性突变。另外47例伴有空洞改变患者中, 10例检测到基因突变, 其中*EGFR*及*KRAS*突变分别为5例(均为50%)。10例基因突变阳性患者中, 1例(5.3%)为鳞癌患者(EGFR外显子19缺失突变); 9例(94.7%)为非鳞癌患者, 其中4例为*EGFR*突变(2例外显子19缺失突变, 2例外显子21 L858R突变)。无空洞改变患者基因突变阳性率为37.7%(63/167), 与有空洞改变患者阳性率(21.3%)比较有显著差异(χ^2^=4.415, *P*=0.036)。无空洞患者*EGFR*突变阳性率为41.0%(50/122), 与有空洞患者*EGFR*阳性率(16.1%)比较, 有显著统计学差异(χ^2^=6.632, *P*=0.010)。Ⅰ期-Ⅲa期患者基因突变率为29.1%(25/86), Ⅲb期-Ⅳ期患者为40.9%(45/110), 尽管晚期肺癌患者基因突变率比早期患者有升高趋势, 但是组间比较无统计学差异(χ^2^=2.947, *P*=0.086)(表 3)。

分析女性、腺癌、不吸烟、无空洞改变、行*EGFR*基因检测的28例患者, 其中高达64.3%(18/28)的患者存在*EGFR*阳性突变, 38.9%(7/18)为外显子19缺失, 50%(9/18)为外显子21 L858R, 11.1%(2/18)为*EGFR*少见突变。

## 3 讨论

来自台湾的一项队列研究发现, 感染肺结核后, 肺癌发病率显著升高, 在肺结核感染后的2年-4年肺癌发病率是无肺结核感染人群的1.98倍, 5年-7年是1.42倍, 8年-12年是1.59倍^[3]^。另一来自沈阳的队列研究^[4]^认为, 感染肺结核后肺癌发病率显著增加, 在感染肺结核20年后, 肺癌的发病率率仍然比未感染肺结核人群高2倍。

本研究中, 肺癌合并肺结核的患者中, 男性为77.3%, 女性为22.7%, 男女发病比例为3.4:1。目前关于肺癌合并肺结核与性别关系的报道, 基本一致, 均认为男性发病率高于女性^[3, 5, 6]^, 分析患者吸烟史发现, 85.3%的男性患者有吸烟史, 女性患者中有吸烟史者为15.2%, 肺结核病及吸烟均为肺癌的高危发病因素^[7]^, 因此可以解释本研究中男性肺癌合并肺结核患者显著多于女性的原因。

局部晚期及进展期肺癌患者, 合并活动性肺结核的发病率为26.4%, 此比例显著高于早期肺癌患者(14.1%)(*P*=0.004)。分析其原因, 可能由于肺癌晚期患者身体免疫功能降低, 因此活动性肺结核比例增加。

目前关于肺癌合并肺结核病理类型的报道, 国内已有报道多以鳞癌为主^[6, 8, 9]^。但事实上, 目前我国肺癌的主要病理类型已经不是鳞癌, 而是腺癌^[10]^。本研究中, 肺结核合并肺癌患者的病理类型同样以腺癌多见, 肺腺癌及肺鳞癌的比例分别为50.1%及32.3%, 尤其在胸部影像学无空洞改变的患者中, 肺腺癌比例高达54.4%, 但是当胸部影像有空洞改变时, 肺鳞癌成为主要的病理类型, 增加为45.9%。本研究发现有空洞的患者基因突变阳性率为21.3%, 显著低于无空洞的肺癌合并肺结核患者(37.7%), 其中有空洞患者EGFR基因突变阳性率只有16.1%, 显著低于无空洞患者的EGFR突变率(41.0%)。而发生基因突变的10例患者中, 仅1例为鳞癌患者, 本研究中有空洞患者病理类型以鳞癌为主, 可以解释此类患者EGFR阳性突变率低的原因。

本研究中肺癌合并肺结核患者的*EGFR*基因突变率35.9%, 外显子19缺失突变及外显子21 L858R突分别为41.8%及50.9%, 少见突变发生率7.3%。目前关于*EGFR*基因突变的研究, 发现亚裔与高加索人有不同, 亚裔和我国肺腺癌患者*EGFR*基因敏感突变率为40%-50%^[11, 12]^, 女性患者基因突变率为50.0%, 显著高于男性患者(27.9%)。有吸烟史患者基因突变率为28.4%, 明显低于无吸烟史者。腺癌患者基因突变率为45.2%, 明显高于鳞癌患者7.1%的基因突变率。

本研究提示, 患者的基因突变率在合并活动性或陈旧性结核组间无差异(*P*=0.357)。尽管Ⅰ期-Ⅲa期患者基因突变率为29.1%, 低于Ⅲb期-Ⅳ期患者40.9%的基因突变率, 但组间比较无统计学差异(*P*=0.086)。

总之, 肺结核是肺癌发病的高危因素之一^[13]^, 肺癌合并肺结核的患者中, 男性发病率约为女性的3倍-4倍, 男性患者中有吸烟史的比例显著高于女性, 此现象或许进一步支持戒烟对于男性的重要性。稳定性肺结核患者并发肺癌时, 肺癌的早期诊断, 可以减少活动性肺结核的发病比例。肺癌合并肺结核患者以肺腺癌多见, 当胸部影像学存在空洞改变时, 肺鳞癌成为主要病理类型。两病并存时, 女性患者基因突变阳性率显著高于男性, 其中腺癌、不吸烟、胸部影像学无空洞改变的女性患者, 其*EGFR*阳性突变率高达63.4%。我们尚需要更多的了解肺癌合并肺结核患者的临床特征、治疗中肺结核及肺癌疾病的转归情况、治疗副反应与单纯肺结核或者肺癌患者的异同, 从而制定出更适合肺癌合并肺结核患者的临床方案。
